# Meta-analysis of FOLFIRINOX-based neoadjuvant therapy for locally advanced pancreatic cancer

**DOI:** 10.1097/MD.0000000000024068

**Published:** 2021-01-22

**Authors:** Zhiliang Chen, Yongshuang Lv, He Li, Rui Diao, Jian Zhou, Tianwu Yu

**Affiliations:** Department of Hepatobiliary Surgery, Yongchuan Hospital, Chongqing Medical University, Yongchuan, Chongqing, China.

**Keywords:** FOLFIRINOX, pancreatic cancer, resection

## Abstract

Currently, the combination of 5-fluorouracil, leucovorin, irinotecan, and oxaliplatin (FOLFIRINOX) is the standard therapy for metastatic pancreatic cancer. In recent years, FOLFIRINOX-based neoadjuvant therapy for locally advanced pancreatic cancer (LAPC) has been gaining an increasing amount of attention, owing to its ability to reduce disease stage and transform LAPC to borderline resectable or even resectable pancreatic cancer. Accordingly, we aimed to evaluate the efficacy of first-line FOLFIRINOX chemotherapy in patients with LAPC.

We searched PubMed, Embase, and Cochrane Library from the time of establishment till January 1, 2020 and included studies focusing on LAPC patients who received FOLFIRINOX as first-line neoadjuvant treatment. The primary outcomes were: resection rate and radical (R0) resection rate while the secondary outcomes were: objective response rate, overall survival, progression-free survival, and rate of grade 3 to 4 adverse events. The meta package for R 3.6.2 was used for heterogeneity and publication bias testing.

Twenty-one studies, including 653 patients with LAPC, were selected. After treatment with FOLFIRINOX, the resection rate was 26% (95% confidence interval [CI] = 20%–32%, I2 = 61%) and R0 resection rate was 88% (95% CI = 78%–95%, I2 = 62%). The response rate was 34% (95% CI = 25%–43%, I2 = 56%). The median overall survival and progression-free survival durations ranged from 10.0 to 32.7 months and 3.0 to 25.3 months, respectively. The observed grade 3 to 4 adverse events were neutropenia (20.0 per 100 patients, 95% CI = 14%–27%, I2 = 75%), febrile neutropenia (7.0 per 100 patients, 95% CI = 5%–9%, I2 = 42%), thrombocytopenia (6.0 per 100 patients, 95% CI = 5%–8%, I2 = 27%), nausea/vomiting (7.0 per 100 patients, 95% CI = 7%–12%, I2 = 76%), diarrhea (10.0 per 100 patients, 95% CI = 8%–12%, I2 = 38%), and fatigue (9.0 per 100 patients, 95% CI = 7%–11%, I2 = 43%).

FOLFIRINOX-based neoadjuvant chemotherapy has the potential to improve the rates of resection, R0 resection, and median OS in LAPC. Our results require further validation in large, high-quality randomized controlled trials.

## Abstract 2

Supplemental Digital Content is available in the text

## 1 Introduction

Pancreatic cancer (PC) is among the most malignant cancers, characterized by rapid progression, poor prognoses, high postoperative recurrence rates, and 5-year survival rates lower than 5%.^[1]^ PC accounted for 4.5% of all cancer-related deaths in 2018,^[2]^ and is expected to become the second leading cause of cancer-related deaths by 2030.^[3]^ The diagnosis of this disease in the early stages is difficult and only 15% to 20% of patients undergo surgery at first diagnosis.^[4,5]^ However, even after radical surgery, the 5-year survival rate associated with the disease is only 15% to 25%.^[6]^

Neoadjuvant chemotherapy for PC, a hot topic in clinical research, has been shown to improve the prognoses of patients, through reductions in the development rate of tumor lesions, increases in the R0 resection rate, reductions in the rates of vascular invasion and micrometastasis, and decreases in the incidence of postoperative complications.^[7–9]^ The combination of 5-fluorouracil, leucovorin, irinotecan, and oxaliplatin (FOLFIRINOX) is increasingly gaining importance in the treatment of metastatic pancreatic cancer (MPC). A randomized controlled trial (RCT) from the United States (PROD-IGY 4/ACCORD 11) showed that MPC patients treated with FOLFIRINOX had a better objective response rate (ORR) (31.6% vs 9.4%, *P* < .001) and median survival duration (11.1 vs 6.8 months, *P* < .001) than those with gemcitabine monotherapy.^[10]^ In addition, FOLFIRINOX can improve the quality of life of MPC patients compared with gemcitabine alone.^[11]^ Given its efficacy in MPC treatment, FOLFIRINOX is now widely used for patients with LAPC, as it has the potential to reduce the disease stage, transform it to borderline resectable or even resectable pancreatic cancer, and improve the associated surgical resection rate.^[12]^ Furthermore, a meta-analysis also indicated that FOLFIRINOX had an advantage in improving the median overall survival (OS) of patients with LAPC.^[13]^ However, most previous studies on the topic had an insufficient sample size, owing to which definitive conclusions about the efficacy and safety of FOLFIRINOX in LAPC patients could not be drawn.

The aim of the present meta-analysis was to assess the efficacy of FOLFIRINOX as a first-line chemotherapy regimen for patients with LAPC.

## 2 Materials and methods

### 2.1 Search strategy

This meta-analysis was PRISMA-compliant. The ethics committee (Medical Ethics Committee of Yongchuan Hospital, Chongqing Medical University) approved the study. We systematically searched PubMed, EMBASE, and the Cochrane Library for relevant studies published from database establishment to January 1, 2020, including RCTs, clinical controlled studies, and cohort studies, among others. The search terms included “FOLFIRINOX,” “fluorouracil,” “irinotecan,” “oxaliplatin,” “pancreatic cancer,” “pancreatic neoplasm,” “drug combination,” and relevant variants thereof. Only studies published in English were included. There were no restrictions on the population, race, or publication date. Potential eligible studies were identified by searching the references of the selected studies. The search was completed independently by 3 authors. Specific details on the search strategy are presented in Appendix 1, Supplemental Content.

### 2.2 Selection criteria and data extraction

All the included studies focused on the effectiveness of FOLFIRINOX in patients with LAPC. The following selection criteria were applied for the included studies: study design: RCT, cohort study, clinical controlled study, etc, presence of at least 1 patient with LAPC, accurate LAPC diagnosis by imaging or pathology prior to chemotherapy, and use of FOLFIRINOX as the first-line chemotherapy regimen for LAPC. In addition, studies were excluded if they met the following criteria: study design: case report, review, conference abstract, and republication, lack of data on resection rate or R0 resection rate, and insufficient data on the outcome of interest, or impossibility of the calculation of the outcome of interest. Two reviewers assessed the titles and abstracts independently for eligibility, and the full tests were further assessed to check if they met the selection criteria. Disagreements were resolved through discussions with a third reviewer. A predefined data collection form was used for the extraction of data from the selected studies. The primary outcomes were the rates of resection and R0 resection after first-line FOLFIRINOX treatment for LAPC. The secondary outcomes were the rates of response, median OS, median progression-free survival (PFS), and grade 3 to 4 adverse events. Other collected information included that on the first author, year of publication, type of study, total sample size, number of patients treated with FOLFIRINOX, median age, performance status, treatment regimen, tumor stage, median chemotherapy duration, and follow-up duration. The characteristics of the selected studies are shown in Table 1.

**Table 1:** Study characteristic.

Author/year	Country/period of study	Total patients	Median age (years; range)	Stage BRPC	LAPC	MPC	Resection/LAPC	Median OS (month)	Median PFS (month)	Median follow-up (month; range)	NOS
Hosein 2012[19]	France/2008–2011	18	58 (41–73)	4	14	—	6/14	32.7	17.3	36.1 (32.9–38.8)	7
Peddi 2012[20]	US/2009–2012	61	58 (37–72)	4	19	38	4/19	NR	12.4	8.5 (1.5–20.4)	6
Gunturu 2013[21]	US/2010–2011	35	61 (48–77)	—	16	19	2/16	17.3	25.3	33.1 (11.4–49.3)	7
Boone 2013[22]	US/2011–2012	21	59 (42–73)	11	10	—	2/10	NR	NR	NR	6
Faris 2013[23]	US/2010–2012	22	63 (49–78)	—	22	—	5/22	NR	11.7	19.3 (NR)	7
Mahaseth 2013[24]	US/2010–2012	60	63 (36–78	4	20	36	4/20	21.2	11.0w	NR	6
Marthey 2014[25]	France/2010–2012	77	61 (37–79)	—	77	—	28/77	22.0	13.0	15.0 (3.0–31.0)	8
Moorcraft 2014[26]	UK/2010–2013	49	60 (34–76)	9	13	27	2/8	18.4	12.9	20.6 (NR)	7
Hohla 2014[27]	Australia/2010–20112	49	62 (42–76)	—	6	28	2/6	10.0	3.0	Not reached	5
Mellon 2015[28]	US/2009–2014	23	67 (45–85)	2	21	—	5/21	24.0	20.4	14.0 (4.0–46.0)	7
Sadot 2015[29]	US/2010–2013	101	64 (37–81)	—	101	—	31/101	25.0	16.0	12.0 (3.0–37.0)	8
Blazer 2015[30]	US/2011–2013	43	62 (40–81)	18	25	—	11/25	NR	NR	13.3 (4.5–34.8)	8
Chllamma 2016[31]	Canada/2011–2014	102	64 (28–76)	—	36	66	6/36	23.0	11.1	NR	5
Berenboim 2018[32]	Israel/2014–2017	53	66 (66–66)	23	30	—	3/30	NR	NR	17.0 (3.0–38.0)	8
Lee 2018[33]	South Korea/2012–2016	64	63 (30–77)	—	64	—	15/64	17.0	NR	23.1 (15.0–46.1)	8
Ulusakarya 2019[34]	France/2016–2016	37	64 (44–81)	—	18	19	11/18	NR	19.8	23.0 (18.0–29.0)	6
Napolitano 2019[35]	Italy/2014–2019	35	59 (42–70)	—	35	—	14/35	24.0	12.4	NR	6
LiXiang 2018[36]	China/2014–2017	41	62 (44–80)	—	41	—	12/41	19.6	13.0	NR	6
Stein 2016[37]	US/2011–2014	68	63 (46–79)	—	31	37	13/31	26.6	17.8	NR	6
Lakatos 2017[38]	Hungary/2014–2016	32	60 (40–77)	—	32	—	2/32	NR	NR	NR	5
Suker 2018[39]	Netherlands/2012–2014	22	62 (52–67)	—	22	—	2/22	15.4	11.0	NR	6

### 2.3 Assessment of the methodological quality of the included studies

The methodological quality of the included studies was evaluated independently according to the Newcastle–Ottawa scale (NOS) by 2 reviewers.^[14]^ The NOS comprises 3 factors: patient selection, comparability of the study groups, and evaluation of results, including 8 items with a full score of 9. A score lower than 4 is indicative of a low study quality and that higher than 7 reflects a high quality. The NOS scores of the included studies are shown in Table 1. Details on the NOS scale are presented in Appendix 2, Supplemental Content and the specific NOS scores of the included studies are presented in Appendix 3, Supplemental Content.

### 2.4 Statistical analysis

We used the meta package of R 3.6.2 software for the data analysis. The heterogeneity of the included studies was assessed using the *χ*^2^-based Q test and I2 test.^[15,16]^ The level of heterogeneity was considered to be significantly different at I2 > 50% or *P* < .1 in the Q test. According to Cochrane review guidelines, the level of heterogeneity was considered significant at I2 > 50%, and the random effects model was selected. Otherwise, the fixed effects model was used to evaluate the 95% confidence intervals (CIs). Additionally, sensitivity analyses of the surgical resection rates were performed by the removal of each study for the assessment of the quality and stability of the results. *P* < .1 was considered statistically significant. Publication bias was assessed using funnel plots (See Appendix 7, Supplemental Content.

## 3 Results

### 3.1 Literature search and study characteristics

**Figure 1:**
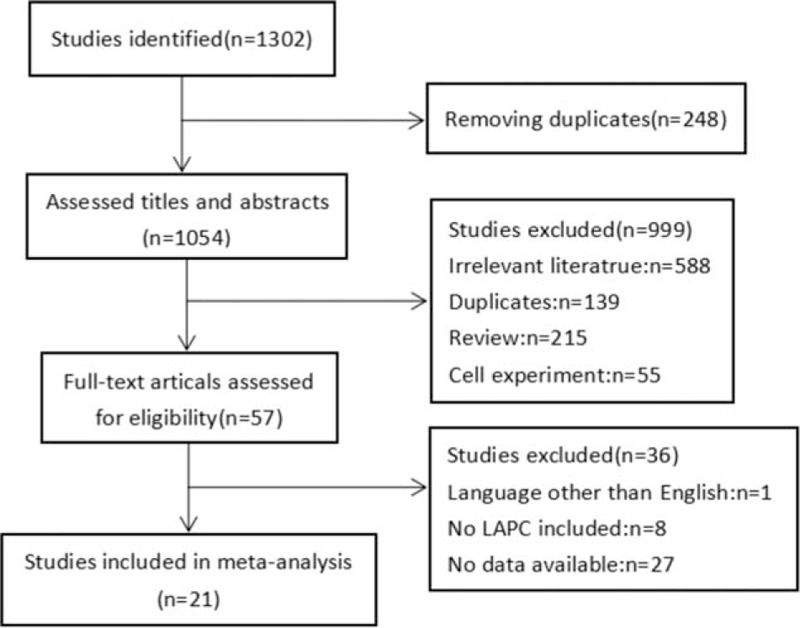
Flow diagram of literature search and study selection.

A total of 1302 studies were identified through the database search; 248 duplicate references were removed and 999 references were excluded after the titles and abstracts were read. The remaining 57 full-text articles were further assessed for eligibility. Finally, 21 articles were included in the meta-analysis.^[19–39]^ A flow chart of the literature search is shown in Figure 1.

The characteristics of the included studies are shown in Table 1. A total of 1013 patients were included from the 21 studies, including 75 patients with borderline resectable pancreatic cancer (BRPC), 270 with MPC, 653 with LAPC, and 15 with tumor recurrence. A majority of the patients had an Eastern Cooperative Oncology Group (ECOG) performance status score of 0 or 1. Overall, 51.0% of the patients were male, 44.6% were female, and 4.4% had an unknown gender. Nearly half of the patients (44.7%) were from the United States. All the studies reported the median age of the patients, which ranged from 58 to 67 years, with the youngest patient aged 34 years and oldest 81 years. One study was a phase II multicenter study, 3 were prospective cohort studies, and 17 were retrospective cohort studies. Eight studies focused on LAPC, 5 on BRPC and LAPC, 4 on LAPC and MPC, 3 on BRPC, LAPC, and MPC, and 1 on LAPC, MPC, and tumor recurrence. LAPC was defined by National Comprehensive Cancer Network (NCCN) criteria in 9 studies,^[17]^ Americas Hepato-Pancreato-Biliary Association/Society of Surgical Oncology/Society for Surgery of the Alimentary Tract (AHPBA/SSO/SSAT) criteria in 3 studies,^[18]^ and other criteria in 9 studies. Sixteen studies reported the median number of FOLFIRINOX cycles administered to patients with LAPC, which ranged from 4.9 to 11.5. And of these studies, 12 reported the median number of FOLFIRINOX cycles was 6 or higher. In the 12 studies, 280 patients received additional radiation after chemotherapy, and in 6 of these, 146 patients received it in combination with gemcitabine, oxaliplatin, capecitabine, or 5-fluorouracil as a radiosensitize, Thirty-one patients from 2 studies were administered stereotactic radiotherapy, of whom 12 patients received a dose of 36 Gy in 3 fractions and the other 19 received a dose of 33 Gy in 5 fractions. Furthermore, 6 patients in 1 study received intraoperative radiotherapy.

### 3.2 Resection and R0 resection rates

**Figure 2:**
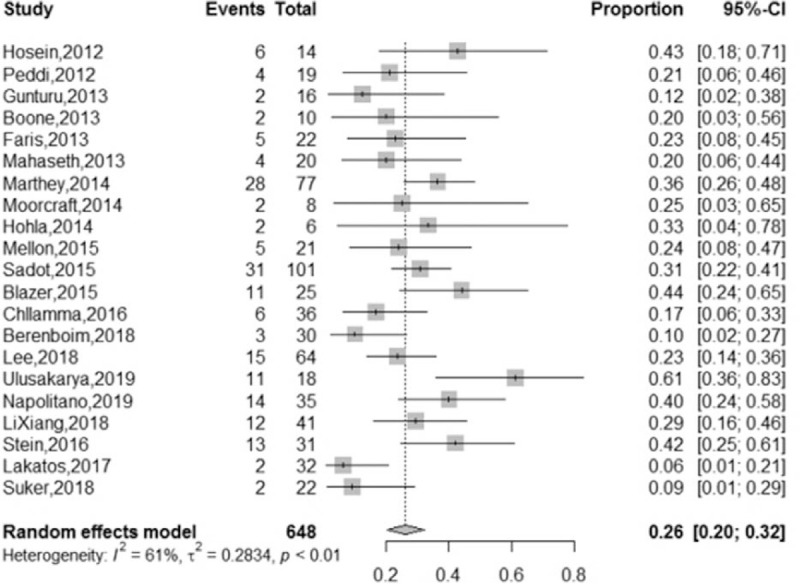
Forest plots of LAPC patients who underwent resection. LAPC = locally advanced pancreatic cancer.

**Figure 3:**
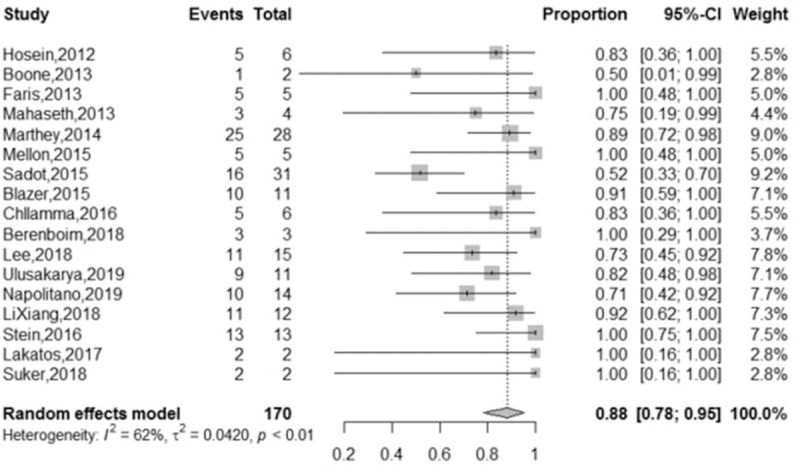
Forest plots of LAPC patients who underwent R0 resection. LAPC = locally advanced pancreatic cancer.

A total of 648 LAPC patients from 21 studies were analyzed. In total, 190 (29%) LAPC patients underwent surgical resection after first-line FOLFIRINOX chemotherapy ± radiotherapy, and the pooled resection rate was 26% (95% CI = 20%–32%, I2 = 61%), with a random-effects model (Fig. 2). Additionally, 126 (74%) of the 170 LAPC patients who underwent surgical resection in 17 studies achieved R0 resection, and the pooled R0 resection rate was 88% (95% CI = 78%–95%, I2 = 62%), using a random-effects model (Fig. 3). We performed subgroup analyses of the rates of surgical resection and R0 resection using the median number of FOLFIRINOX cycles as a grouping factor. In 12 studies, in which the median number of FOLFIRINOX cycles was 6 or lower, the surgical resection rate was 26% (95% CI = 19%–34%, I2 = 61%) compared with the 34% (95% CI = 27–43, I2 = 0%) observed in 4 studies, in which the median number of cycles was lower than 6 (*P* = .12). Meanwhile, the R0 resection rate in 10 studies, in which the patients received 6 or fewer median FOLFIRINOX cycles was 90% (95% CI = 74%–99%, I2 = 69%) compared with the 88% (95% CI = 77%–96%, I2 = 0%) observed in 3 studies in which the median number of cycles was lower than 6 (*P* = .81). All the studies used a random-effects model and the forest plots are shown in Figures 1A and 2B in Appendix 4, Supplemental Content. In addition, we eliminated studies one by one for the performance of sensitivity analyses of the surgical resection rate, and found that there was no directional change in the heterogeneity of the results.

### 3.3 Median OS, median PFS, and ORR

We obtained data on the median OS for LAPC patients from 14 studies, which ranged from 10.0 to 32.7 months. The median PFS of the LAPC patients in 16 studies ranged from 3.0 to 25.3 months (Table 1). Data on the ORR in the LAPC patients treated with FOLFIRINOX were obtained from 8 studies, and ranged from 17.20% to 55.6%; the pooled ORR was 34% (95% CI = 25%–43%, I2 = 56%) (Fig. 2 in Appendix 5, Supplemental Content.

### 3.4 Grade 3 to 4 adverse events

A total of 19 studies reported grade 3 to 4 adverse events during treatment with FOLFIRINOX, but one of them reported only the total number of outcomes. In these studies, 875 patients were treated with FOLFIRINOX and 570 grade 3 or 4 adverse events were reported (65.1 events per 100 patients). Only 1 study reported a toxic death attributed to FOLFIRINOX and the most likely cause of death was pulmonary embolism. The most commonly reported grade 3 to 4 adverse hematological events included neutropenia, febrile neutropenia, and thrombocytopenia (Table 2); the corresponding pooled rates per 100 patients were 20% (95% CI = 14–27%, I2 = 75%), 7% (95% CI = 5–9%, I2 = 42%), and 6% (95% CI = 5–8%, I2 = 27%), respectively. The most commonly observed grade 3-4 non-hematological adverse events were fatigue, nausea/vomiting, and diarrhea (Table 3), the pooled rates per 100 patients of which were 9% (95% CI = 7%–11%, I2 = 43%), 7% (95% CI = 7%–12%, I2 = 76%), and 10% (95% CI = 8%–12%, I2 = 38%), respectively (Fig. 3 in Appendix, Supplemental Content. A majority of the adverse events could be effectively controlled by systematic treatment or reductions in the chemotherapy drug dose.

**Table 2:** G3 to G4 adverse events^∗^ of hematologic.

Author	Total number	Neutr-openia	Febrile neutropenia	Anemia	Throm-bocytopenia	Infections
Hosein	12	4	3	2	3	—
Peddi	17	12	3	—	2	—
Gunturu	6	4	1	—	1	—
Boone	5	3	—	—	2	—
Faris	5	4	—	—	1	—
Mahaseth	8	2	—	—	3	3
Marthey	10	9	—	1	—	—
Moorcraft	28	14	7	2	5	—
Blazer	—	—	—	—	—	—
Chllamma	49	38	6	3	2	—
Berenboim	3	—	1	—	2	—
Lee	50	28	10	9	3	—
Ulusakarya	—	—	—	—	—	—
Napolitano	11	10	—	—	1	—
Stein	23	9	3	4	7	—
Lakatos	23	9	1	8	5	—
Suker	1	—	1	—	—	—
LiXiang	25	10	1	9	5	—

**Table 3:** G3 to G4 adverse events^∗^ of nonhematologic.

Author	Total	Fatigue	Vomiting/nausea	Diarrhea	Neuropath	Abdom-inal pain	Elevated ALT and AST	Thromb-o embolism	Others
Hosein	4	2	—	2	—	—	—	—	—
Peddi	10	3	—	2	—	5	—	—	—
Gunturu	4	2	1	1	—	—	—	—	—
Boone	7	—	—	1	1	—	—	—	5
Faris	3	—	—	—	—	—	2	1	—
Mahaseth	27	8	5	8	3	—	—	—	3
Marthey	20	5	7	5	3	—	—	—	—
Moorcraft	31	9	4	2	2	—	—	6	8
Blazer	16	4	2	6	—	—	—	—	4
Chllamma	53	1	35	16	—	—	—	—	1
Berenboim	—	—	—	—	—	—	—	—	—
Lee	30	7	12	8	3	—	—	—	—
Ulusakarya	9	5	1	2	1	—	—	—	—
Napolitano	4	—	1	2	—	—	1	—	—
Stein	31	9	2	12	2	—	3	3	—
Lakatos	14	4	6	4	—	—	—	—	—
Suker	12	1	1	4	—	—	3	—	3
LiXiang	5	—	1	1	—	—	2	1	—

## 4 Discussion

In our meta-analysis, which included 21 studies involving 648 patients with LAPC who received FOLFIRINOX as first-line chemotherapy, we observed an ORR of 34% (95% CI = 25%–43%, I2 = 56%), which was significantly higher than the value associated with gemcitabine treatment (9.4%).^[10]^ In addition, previous studies have shown that objective efficiency can improve patients’ prognoses and survival, suggesting that the FOLFIRINOX approach rather than the gemcitabine-based approach may have potential benefits, in terms of survival outcomes.^[40,41]^

PC is systemic in nature and up to 85% of those with the disease are diagnosed with tumors that involve local arteries or distant metastases^[42,43]^; therefore, palliative chemotherapy has become the mainstay in the treatment of advanced PC. In 1997, a randomized trial by Burris et al^[44]^ confirmed that patients receiving gemcitabine monotherapy had a slight advantage in terms of median OS over those receiving 5-fluorouracil monotherapy in LAPC and MPC settings (5.6 vs 4.4 months, *P* < .001). In the years that followed, gemcitabine became the standard treatment for MPC and LAPC. Chauffert et al^[45]^ evaluated gemcitabine as a first-line treatment for LAPC, and showed a median OS duration of 6 to 13 months. However, in 2011, an RCT showed that FOLFIRINOX treatment improved the PFS (6.4 vs 3.3 months) and OS (11.1 vs 6.8 months) durations in MPC patients to a greater degree than gemcitabine monotherapy.^[10]^ As a majority of LAPC patients show better performance rates than MPC patients, a growing number of studies are now using FOLFIRINOX as the first-line chemotherapy regimen for patients with LAPC. Suker et al^[13]^ conducted a patient-level meta-analysis that evaluated the role of FOLFIRINOX in LAPC patients, including 11 studies involving 315 patients, and demonstrated a median OS duration of 24.0 months (95% CI = 21.7–26.8 months). Subsequently, Suker et al^[39]^ conducted a cohort study that contrasted with the gemcitabine scheme investigated by Chauffert et al, and showed that FOLFIRINOX treatment in LAPC patients resulted in longer median OS durations; their work significantly contributed to the use of FOLFIRINOX in such settings. It follows that LAPC patients have longer survival durations than MPC patients after treatment with FOLFIRINOX. Compared with the values observed by Chauffert et al, our meta-analysis of 21 studies reported median OS values ranging from 10.0 to 32.7 months and median PFS values ranging from 3.0 to 25.3 months in LAPC patients. Our results indicate that FOLFIRINOX exhibits stronger efficacy than gemcitabine in LAPC patients.

PC is associated with high mortality values, and most patients with early-stage disease tend to die. Surgical resection is the only chance for cure in PC, although few patients are eligible for surgery. In a previous meta-analysis of PC patients who underwent surgical resection, a survival duration of 3 to 5 years was observed, which was longer than that noted among those who did not undergo surgical resection.^[46]^ Therefore, for a large number of patients with LAPC, surgical resection yields the highest long-term values.^[47]^ In the past, only 1% to 5% of LAPC patients underwent complete surgical resection after single-drug neoadjuvant chemotherapy.^[48,49]^ Fortunately, a growing number of studies are now reporting that LAPC patients who receive neoadjuvant FOLFIRINOX may potentially be able to undergo surgical resection as a result of tumor downstaging. All the 21 studies included in the current meta-analysis reported surgical resection rates ranging from 6.3% to 61.1% in LAPC patients with neoadjuvant FOLFIRINOX chemotherapy. The surgical resection rates reported across different studies show an obvious degree of heterogeneity. At the same time, in our sensitivity analysis of the rate of surgical resection, we found that when 2 studies were excluded (Ulusakarya et al and Lakatos et al), the level of heterogeneity of the results decreased, but there was no directional change. One reason for this difference may be the lack of consensus regarding the resectability criteria after neoadjuvant therapy. Therefore, for the performance of more accurate comparisons, future studies may need to reach a consensus on the resectability criteria. In the present meta-analysis, the surgical resection rate in the LAPC patients with FOLFIRINOX as first-line chemotherapy was 26% and 88% of these patients underwent R0 resection. The R0 resection rate was even higher than that reported by Gillen et al^[50]^ in patients with a resectable status (88% vs 80%). This indicates that the use of the FOLFIRINOX regimen significantly increases the chance of R0 resection in LAPC patients. In addition, we performed subgroup analyses of the surgical resection rates and R0 resection rates using the median number of FOLFIRINOX cycles as a grouping factor to evaluate whether the number of FOLFIRINOX cycles influences the rates of surgical resection and R0 resection; however, no statistically significant differences were observed. This may be attributed to the small sample size of the included studies. In addition, Janssen et al^[51]^ showed that a median number of FOLFIRINOX cycles were greater than or equal to 6, with a median OS of 21.4 months (95% CI = 16.7–36.0 months), compared with those observed of 21.7 months (95% CI = 15.0–28.4 months) in a study with a median number of chemotherapy cycles lower than 6, with no statistical difference. Therefore, further studies are required to investigate whether the median number of FOLFIRINOX cycles is an important factor in the determination of the rates of surgical resection and median OS.

In addition, in terms of the rate of local tumor progression, we found that 28% of the patients received additional radiotherapy or chemoradiotherapy after FOLFIRINOX treatment. There was no significant difference in the degree of resectability between patients who received radiotherapy or chemoradiotherapy and those who did not. A meta-analysis of 14 phase II clinical trials showed that the rates of surgical resection and R0 resection were 32% and 20%, respectively, in LAPC patients after neoadjuvant chemotherapy, with or without radiotherapy.^[52]^ Meanwhile, in another recently published study, 26% of 215 patients with LAPC who received chemoradiotherapy underwent surgical resection and 10% achieved R0 resection.^[53]^ Thus, although the use of radiotherapy or chemoradiotherapy may be convincing for local control, the unresectable status persists after treatment in many patients. Therefore, the role of these treatments in locally advanced disease needs further clarification in future studies.^[54]^

Although the effect of FOLFIRINOX is superior to that of gemcitabine in patients with metastatic PC, its toxicity somewhat hinders its clinical application. In our study, the most commonly observed grade 3 to 4 adverse event was neutropenia, which showed an incidence that was similar to that reported by Suker et al (20% vs 19.6%). Granulocyte-colony stimulating factor is widely used in the prevention of this hematological toxicity. In addition, in a majority of the studies we included, the dose of the chemotherapeutic drugs was reduced based on the patients’ tolerance level, with the aim of reducing the rates of adverse events and improving the efficacy of chemotherapy. It has been demonstrated that the modified FOLFIRINOX regimen yields satisfactory results in patients with an ECOG performance status score of 0 to 1.^[55]^

This study has some limitations that should be considered. First, the sample size of the included studies was relatively small, with a high level of heterogeneity, and a majority of the studies had a retrospective design (17/21, 81%), which may challenge the accuracy of the study results and lead to their overestimation. Second, different criteria were used in the diagnosis of LAPC. Most of the studies referred to NCCN or AHPBA/SSO/SSAT criteria, while some used other criteria, affecting the determination of the outcome indicators. Third, a majority of the studies did not clearly report the implementation details of the FOLFIRINOX-based treatment regimens and involved reductions in the dose of the chemotherapeutic drugs. The presence of heterogeneity across the studies may have biased the results. Fourth, the included studies did not report on the ethnicities and dietary habits of the study populations, owing to which the comprehensiveness of the results is low.

In conclusion, FOLFIRINOX-based neoadjuvant chemotherapy can improve the rates of resection, R0 resection, and median OS in LAPC. These results require further validation in large, high-quality RCTs.

## Author contributions

**Conceptualization:** Tianwu Yu.

**Data curation:** Zhiliang Chen, Rui Diao.

**Formal analysis:** Zhiliang, Chen, Yongshuang, Lv.

**Methodology:** Yongshuang Lv, Jian, Zhou.

**Software:** Zhiliang, Chen.

**Supervision:** Tianwu, Yu.

**Writing – original draft:** Zhiliang, Chen, Yongshuang, Lv.

**Writing – review & editing:** He Li, Tianwu Yu.

## Supplementary Material

Supplemental Digital Content
