# Supplementary material for: Meta-analysis of FOLFIRINOX-based neoadjuvant therapy for locally advanced pancreatic cancer
Source: Medicine (Baltimore). 2021 Jan 22;100(3):e24068. doi: 10.1097/MD.0000000000024068 (PMC7837836; doi:10.1097/MD.0000000000024068)
Supplement: Supplemental Digital Content [file medi-100-e24068-s001.pdf]

Supplemental Content

Meta-analysis of FOLFIRINOX-based neoadjuvant therapy for locally advanced pancreatic cancer

Chen,Zhiliang;Lv,Yongshuang;Li,He;Diao,Rui;Zhou,Jian;Yu,Tianwu

Appendix1

Search strategy

Database		Search for	hits
PubMed	pubmed	(Folfirinox[Title/Abstract]) AND Pancreatic Neoplasms/therapy[Mesh]	410
Embase	all fields	(Folfirinox:ti,ab) AND (drug AND combination) AND ((pancreatic cancer:ti,ab) or (pancreatic tumor:ti,ab) or (pancreatic neoplasms:ti,ab))	773
The Cochrane library	search all text	(Folfirinox):ab,ti	109
**No. (Total)**			**1302**

Appendix2

NEWCASTLE - OTTAWA QUALITY ASSESSMENT SCALE COHORT STUDIES

Selection

1) Representativeness of the exposed cohort

a) truly representative of the average of the exposed cohort in the community ★

b) somewhat representative of the average the exposed cohort in the community ★

c) selected group of users eg nurses, volunteers

d) no description of the derivation of the cohort

2) Selection of the non exposed cohort

a) drawn from the same community as the exposed cohort ★

b) drawn from a different source

c) no description of the derivation of the non exposed cohort

3) Ascertainment of exposure

a) secure record (eg surgical records) ★

b) structured interview ★

c) written self report

d) no description

4) Demonstration that outcome of interest was not present at start of study

a) yes ★

b) no

Comparability

1) Comparability of cohorts on the basis of the design or analysis

a) study controls for the most important factor ★

b) study controls for any additional factor ★

Outcome

1) Assessment of outcome

a) independent blind assessment ★

b) record linkage ★

c) self report

d) no description

2) Was follow-up long enough for outcomes to occur

a) yes(more than 1 year) ★

b) no

3) Adequacy of follow up of cohorts

a) complete follow up - all subjects accounted for ★

b) subjects lost to follow up unlikely to introduce bias - small number lost - > 80% ★

c) follow up rate < 80% and no description of those lost

d) no statement

Note: A study can be awarded a maximum of one star for each numbered item within the Selection and Outcome categories. A maximum of two stars can be given for Comparability

Appendix3

the specific NOS scores of the included studies

Studies							NOS
Hosein	Peddi	Gunturu	Boone	Faris	Mahaseth	Marthey	
N	N	N	N	N	N	N	Truly representative of the average of the exposed cohort in the community

Y	Y	Y	Y	Y	Y	Y	Somewhat representative of the average the exposed cohort in the community
N	N	N	N	N	N	N	Drawn from the same community as the exposed cohort
Y	Y	Y	Y	Y	Y	Y	Ascertain the exposure by secure record
N	N	N	N	N	N	N	Ascertain the exposure by structured interview
Y	Y	Y	Y	Y	Y	Y	Demonstration that outcome of interest was not present at start of study
N	N	N	N	N	N	Y	Study controls for the most important factor
Y	Y	Y	Y	Y	Y	Y	Study controls for any additional factor
N	N	N	N	N	N	N	Assessment of outcome by independent blind assessment
Y	Y	Y	Y	Y	Y	Y	Assessment of outcome by record linkage
Y	N	Y	N	Y	N	Y	Median follow-up long enough for outcomes to occur (more than 10 month)
Y	Y	Y	N	Y	Y	Y	Complete follow up - all subjects accounted for
N	N	N	Y	N	N	N	Subjects lost to follow up unlikely to introduce bias (follow-up rate > 80%)

Studies								NOS
Moorcraft	Hohla	Mellon	Sadot	Blazer	Chllamma	Berenboim	Lee	
N	N	N	N	N	N	N	N	Truly representative of the average of the exposed cohort in the community

Y	Y	Y	Y	Y	Y	Y	Y	Somewhat representative of the average the exposed cohort in the community
N	N	N	N	N	N	Y	Y	Drawn from the same community as the exposed cohort
Y	Y	Y	Y	Y	Y	Y	Y	Ascertain the exposure by secure record
N	N	N	N	N	N	N	N	Ascertain the exposure by structured interview
Y	Y	Y	Y	Y	Y	Y	Y	Demonstration that outcome of interest was not present at start of study
N	N	N	Y	Y	N	N	N	Study controls for the most important factor
Y	Y	Y	Y	Y	Y	Y	Y	Study controls for any additional factor
N	N	N	N	N	N	N	N	Assessment of outcome by independent blind assessment
Y	Y	Y	Y	Y	Y	Y	Y	Assessment of outcome by record linkage
Y	N	Y	Y	Y	N	Y	Y	Follow-up long enough for outcomes to occur (more than 10 month)
Y	N	Y	Y	N	N	Y	Y	Complete follow up - all subjects accounted for
N	N	N	N	Y	N	N	N	Subjects lost to follow up unlikely to introduce bias (follow-up rate > 80%)

Studies						NOS
Ulusakarya	Napolitano	LiXiang	Stein	Lakatos	Suker	
N	N	N	N	N	N	Truly representative of the average of the exposed cohort in the community
Y	Y	Y	Y	Y	Y	Somewhat representative of the average the exposed cohort in the community

N	Y	N	N	N	N	Drawn from the same community as the exposed cohort
Y	Y	Y	Y	Y	Y	Ascertain the exposure by secure record
N	N	N	N	N	N	Ascertain the exposure by structured interview
Y	Y	Y	Y	Y	Y	Demonstration that outcome of interest was not present at start of study
N	N	Y	Y	N	N	Study controls for the most important factor
Y	Y	Y	Y	Y	Y	Study controls for any additional factor
N	N	N	N	N	N	Assessment of outcome by independent blind assessment
Y	Y	Y	Y	Y	Y	Assessment of outcome by record linkage
Y	N	N	N	N	N	Follow-up long enough for outcomes to occur (more than 10 month)
N	N	N	N	N	Y	Complete follow up - all subjects accounted for
Y	N	N	N	N	N	Subjects lost to follow up unlikely to introduce bias (follow-up rate > 80%)

Appendix4

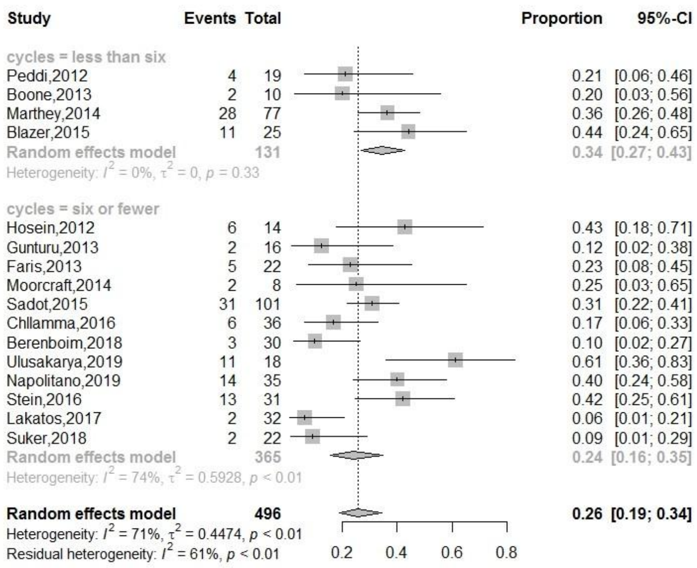

Figure 1A:Forest plots showing the resection rates for studies in which patients received a median number of FOLFIRINOX cycles of six or fewer and those received a median of less than six cycles(P=.12).

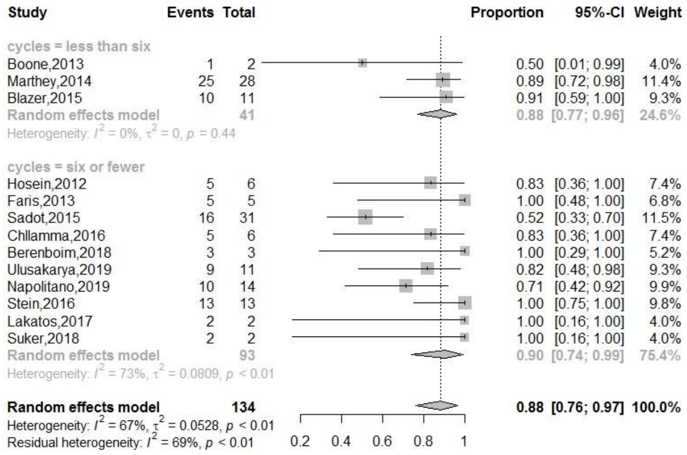

Figure 1B:Forest plots showing the R0 resection rates for studies in which patients received a median number of FOLFIRINOX cycles of six or fewer and those received a median of less than six cycles(P=.81).

Appendix5

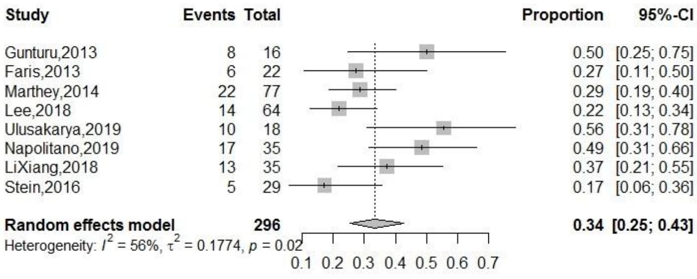

Figure 2 Objective response rates for LAPC patients treated with FOLFIRINOX

Appendix6

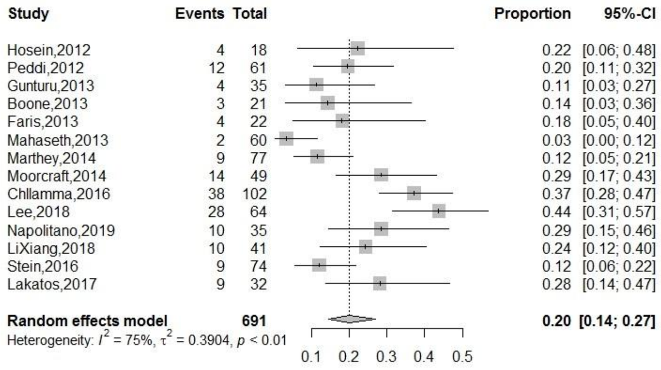

Figure 3A Grade 3 to 4 adverse events for LAPC patients treated with FOLFIRINOX(Neutropenia)

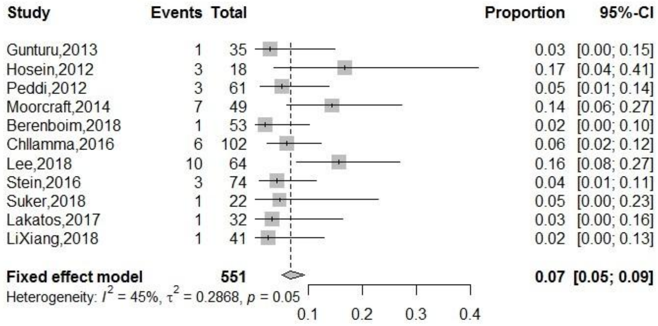

Figure 3B Grade 3 to 4 adverse events for LAPC patients treated with FOLFIRINOX(Febrile neutropenia)

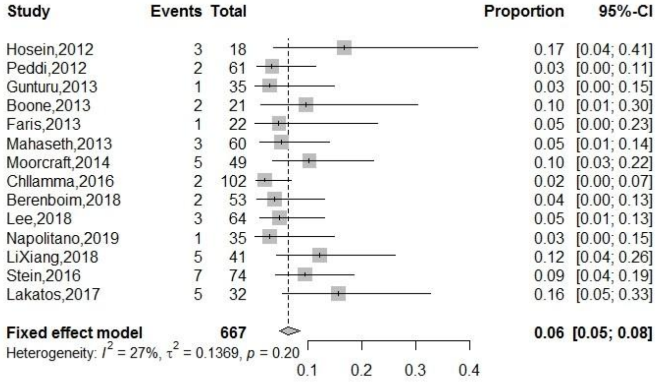

Figure 3C Grade 3 to 4 adverse events for LAPC patients treated with FOLFIRINOX(Thrombocytopenia)

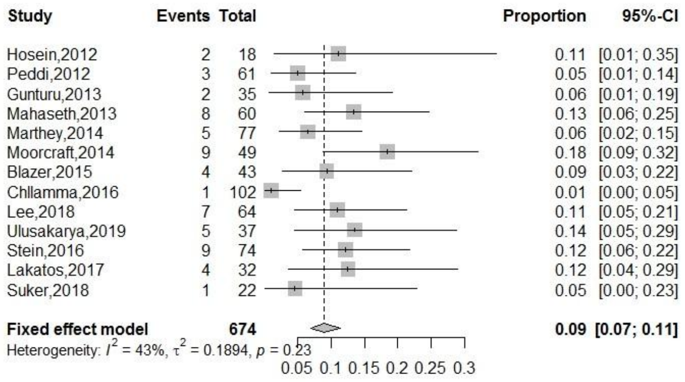

Figure 3D Grade 3 to 4 adverse events for LAPC patients treated with FOLFIRINOX(Fatigue)

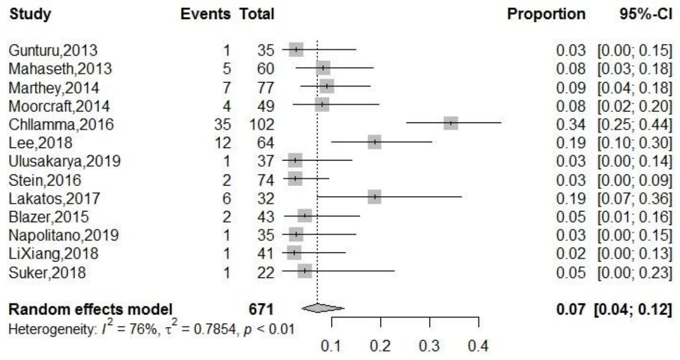

Figure 3E Grade 3 to 4 adverse events for LAPC patients treated with FOLFIRINOX(Nausea/vomiting)

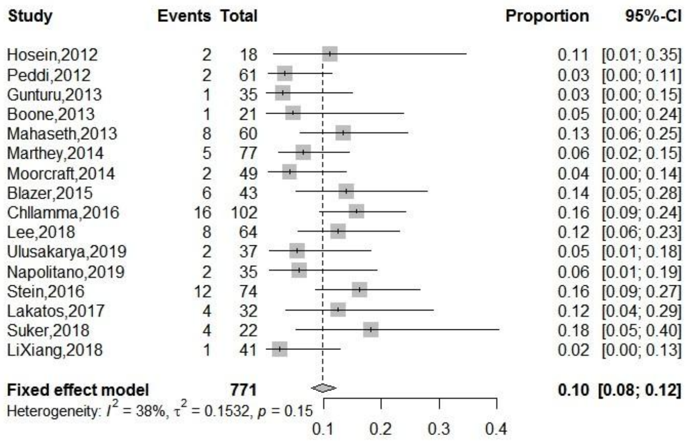

Figure 3F Grade 3 to 4 adverse events for LAPC patients treated with FOLFIRINOX(Diarrhea)

Appendix7

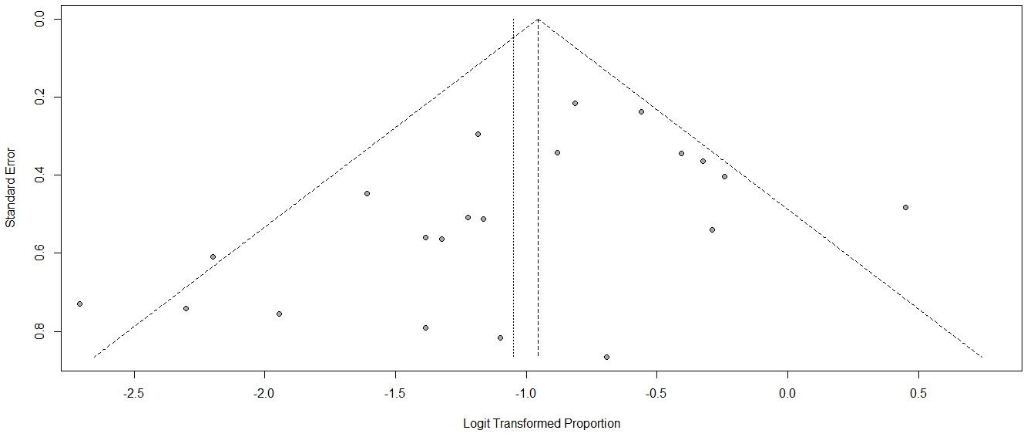

Figure 4 Funnel plot of the resection rates of studies(all studies were shown in this funnel plot)
